# Sacubitril/valsartan in heart failure: a critical look at dementia risk and future study pathways

**DOI:** 10.1093/ehjcvp/pvaf080

**Published:** 2025-10-31

**Authors:** Weikai Dong, Wei Li

**Affiliations:** Department of Cardiovascular Surgery, Binzhou Medical University Hospital, No.661, Yellow River Second Road, Binzhou, Shandong Province 256600, China; Department of Cardiovascular Surgery, Binzhou Medical University Hospital, No.661, Yellow River Second Road, Binzhou, Shandong Province 256600, China

To the Editor:

We have read with great interest the recent publication by Jung K *et al.*^1^ entitled ‘Association between sacubitril/valsartan and risk of dementia in patients with heart failure: a nationwide cohort study’.^1^ The study explored the potential association between sacubitril/valsartan and dementia risk among patients with HF using nationwide data from South Korea. The work is commendable for its large sample size, robust propensity-score matching, and the inclusion of a clinically meaningful lag period. However, several methodological and interpretative aspects warrant further discussion.

Firstly, the authors added an extensive list of comorbidities and medications to their propensity-score model but were unable to adjust for clinically relevant biomarkers such as LVEF, NT-proBNP, and eGFR due to limitations inherent to the data source. These biomarkers are not only essential for HF phenotyping but also strong predictors of adverse outcomes—including cognitive impairment and hospitalization—in patients with concomitant Alzheimer’s disease and HF.^2^ Therefore, while the authors adjusted for numerous measured variables, some clinically meaningful factors remained unaccounted for. Because lower eGFR and elevated NT-proBNP are established predictors of incident dementia, future investigations should integrate laboratory and imaging parameters to enhance confounding adjustment and diagnostic precision.

Secondly, the authors used administrative claims to identify dementia diagnoses based on diagnostic codes and antidementia prescriptions. Although these measures have a high positive predictive value, they cannot capture subclinical cognitive decline and offer limited ability to differentiate specific dementia aetiologies. Notably, population-based data from Denmark have highlighted the limitations of registry-based dementia identification and underscored the need for clinical validation through neuropsychological assessment or neuroimaging.^3^

Thirdly, the gender distribution in the study was highly skewed (∼71% male), which may affect generalizability. Previous nationwide studies have reported sex-specific associations between HF and dementia risk, showing that women have a significantly higher incidence of dementia, which may indicate a sex-specific vulnerability.^3^ Therefore, the possible protective effect of sacubitril/valsartan may not be fully generalizable to the broader HF population. Additionally, the follow-up period (mean ∼2.2 years) may be too short to capture the full latency of dementia onset, as neurodegeneration progresses slowly over time.

Furthermore, the observed protective association (HR 0.84; 95% CI 0.72–0.98) was primarily driven by a reduction in overall dementia, while statistical significance was not achieved for Alzheimer’s dementia (HR 0.88; 95% CI 0.75–1.03), which accounted for over 90% of cases. Although these findings should be interpreted cautiously, they remain compatible with a plausible vascular mechanism. While direct neuroprotective effects remain speculative, recent evidence shows that early in-hospital conversion to ARNI therapy significantly improves left ventricular reverse remodeling in acute HF,^4^ supporting a potential cardiac–cerebral link that could underlie the observed association.

Importantly, as acknowledged by Jung K *et al.*^1^ although a 1:4 propensity-score matching approach was applied, residual confounding may still have been present. Experience from large observational studies employing new-user active-comparator designs—such as the investigation by Kim *et al.*^5^ on statins and dementia risk—illustrates how such approaches improve confounding control and provide valuable methodological guidance for future pharmacoepidemiologic research.^5^

In conclusion, the findings are reassuring regarding the cognitive safety of sacubitril/valsartan; however, the observed effect size was modest, relevant covariate data were limited, and the follow-up duration was relatively short. Future prospective studies incorporating cognitive assessments, biomarker data, and diverse populations are warranted to clarify the neurocognitive impact of ARNI therapy in HF. We offer suggestions aimed at refining an already exceptional article and eagerly anticipate future insightful contributions from the authors.

## Data Availability

Data availability is not applicable to this article as no new data were created or analyzed in this study.
